# Sternum-sparing multivessel coronary surgery as a routine procedure: Midterm results of total coronary revascularization via left anterior thoracotomy

**DOI:** 10.1016/j.xjtc.2024.05.018

**Published:** 2024-06-03

**Authors:** Christian Sellin, Ahmed Belmenai, Margit Niethammer, Volker Schächinger, Hilmar Dörge

**Affiliations:** a Department of Cardiothoracic Surgery, Heart-Thorax Center, Klinikum Fulda, University Medicine Marburg, Campus Fulda, Fulda, Germany; b Department of Cardiology, Heart-Thorax Center, Klinikum Fulda, University Medicine Marburg, Campus Fulda, Fulda, Germany

**Keywords:** minimally invasive cardiac surgery, coronary artery bypass grafting, CABG, TCRAT

## Abstract

**Objective:**

A sternum-sparing approach of minimally invasive total coronary revascularization via left anterior thoracotomy demonstrated promising early outcomes in unselected patients with coronary artery multivessel disease. Follow-up data are still missing.

**Methods:**

From November 2019 to September 2023, coronary artery bypass grafting via left anterior minithoracotomy on cardiopulmonary bypass and cardioplegic cardiac arrest was performed as a routine procedure in 392 consecutive, nonemergency patients (345 men; 67.0 ± 9.9 years; range, 32-88 years). All patients had multivessel coronary artery disease (77.6% 3-vessel-disease, 22.4% 2-vessel-disease, and 32.9% left main stenosis). Patients at old age (older than a 80 years, 12.5%), with severe left ventricular dysfunction (ejection fraction <30%, 7.9%), diabetes mellitus (34.9%), massive obesity (body mass index > 35, 8.9%), and chronic lung disease (17.1%) were included. Mean European System for Cardiac Operative Risk Evaluation II score was 2.9 ± 2.8. Mean midterm follow-up (100%) was 15.2 ± 10.7 months (range, 0.1-39.5 months).

**Results:**

Left internal thoracic artery (99.0%), radial artery (70.4%), and saphenous vein grafts (57.4%) were used, and 70.4% of patients received at least 2 arterial grafts. A total of 3.0 ± 0.8 anastomoses (range, 2-5 anastomoses) per patient were performed to revascularize the territories of left anterior descending (98.7%), circumflex (91.6%), and right coronary (70.9%) artery. Complete anatomical revascularization was achieved in 95.1%. At follow-up, all-cause-mortality, myocardial infarction, repeat revascularization, and stroke was 3.1%, 1.5%, 5.4%, and 0.7%, respectively. Overall major adverse cardiac and cerebrovascular events rate was 8.7%.

**Conclusions:**

This is the first report of midterm follow-up after routine sternum-sparing total coronary revascularization via left anterior thoracotomy for multivessel coronary artery disease with a high rate of multiple arterial grafting and complete anatomical revascularization. Outcome was favorable and similar to that of contemporary conventional coronary artery bypass grafting.

## Graphical Abstract

**Figure:**
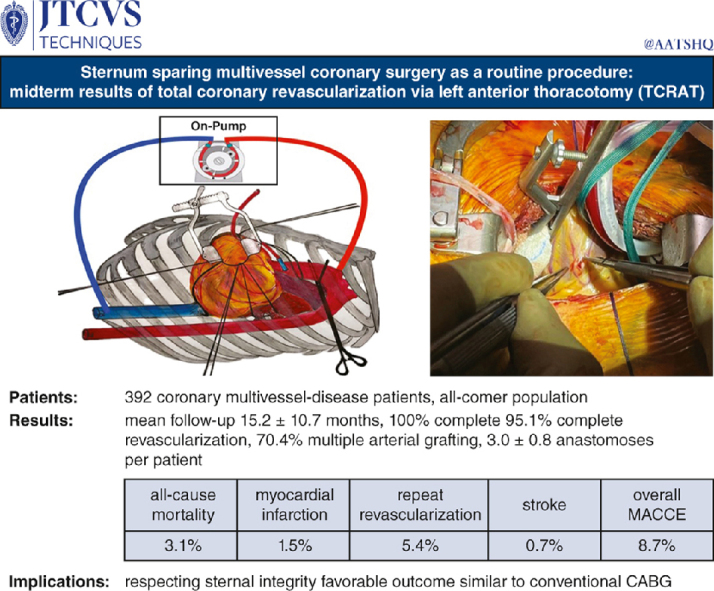

**Figure:**
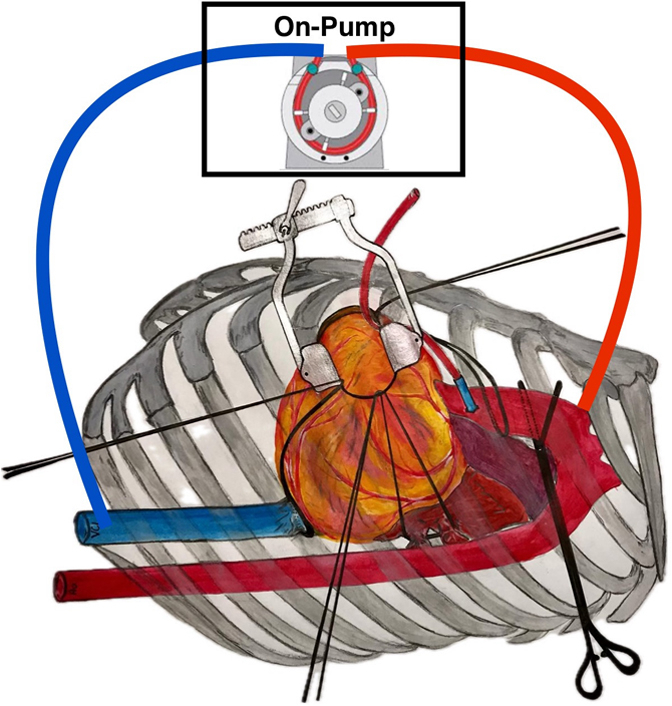
Schematic illustration of on-pump sternum-sparing multivessel coronary surgery.

**Central Message**

The sternum-sparing TCRAT technique allows complete coronary revascularization using predominantly arterial grafts in a majority of patients with multivessel disease with favorable midterm outcome.

**Perspective**

The presented midterm follow-up of sternum-sparing TCRAT is favorable and similar to that of contemporary conventional CABG. Respecting sternal integrity might improve acceptance of surgical coronary revascularization. It is conceivable that long-term results of TCRAT, especially when multiple arterial grafting is used, will be positive, as has been proven for conventional CABG.

Coronary artery bypass grafting (CABG) is the most frequently performed cardiac surgical procedure,^1^ and remains the most robust therapy of coronary revascularization in multivessel coronary disease.^2^ Therefore, CABG is recommended as first-line therapy for complex multivessel coronary disease.^3^ Full midline sternotomy is the standard approach for the overwhelming majority of CABG procedures worldwide.^4^

Different minimally invasive sternum-sparing techniques; for example, robotic endoscopic CABG^5^ and minimally invasive cardiac surgery (MICS) CABG,^6^ have been developed to reduce the invasiveness of conventional CABG with full median sternotomy and the associated limitations with regard to prolonged recovery of physical activity, reduced quality of life, pain, and sternal wound infections. Probably due to technical complexity and enormous infrastructural prerequisites, robotic endoscopic CABG is currently used only in specialized centers and on highly selected patients.^7^ Likewise, MICS CABG has been introduced to revascularize patients with multivessel coronary disease through a small lateral thoracic incision by using special retractors, cardiac apical positioners, and epicardial stabilizers.^6^ This surgically demanding and challenging technique has been routinely implemented into everyday clinical practice by only a few groups worldwide, so that just a very small fraction of patients have undergone MICS CABG so far.^7^ No technique available up to this point has systematically eliminated sternotomy while preserving key principles of complete revascularization and maintaining wide applicability for the broad majority of patients.

In 2019, Babliak and colleagues^8^ proposed a new surgical approach for total coronary revascularization in multivessel coronary artery disease via left anterior minithoracotomy (TCRAT). This technique was further developed by Dörge and colleagues^9^ as a sternum-sparing routine concept of total arterial CABG. The procedure can be applied in unselected patients with promising early results using long-approved surgical methods like cardiopulmonary bypass (CPB), aortic crossclamping, cardioplegic cardiac arrest, and standard anastomosic techniques.^9^^,^^10^ However, currently no follow-up data are available. This report investigated midterm results after CABG in unselected patients with the TCRAT technique.

## Methods

### Patient Selection and Data Collection

From November 2019 to September 2023 a total of 392 consecutive patients underwent nonemergency isolated CABG via left anterior minithoracotomy on CPB with peripheral cannulation and cardioplegic cardiac arrest (transthoracic aortic crossclamping) in our institution, using this technique as a default strategy in daily routine. During this interval, another 11 patients in whom it was intended to perform TCRAT were intraoperatively converted to conventional sternotomy, resulting in a 2.8% conversion rate. Reason for conversion to sternotomy were severe bleeding in 5 patients (ascending aorta n = 2, superior vena cava n = 1, inferior vena cava n = 1, and pulmonary trunk n = 1), circulatory instability due to acute myocardial ischemia during graft preparation in 3 patients, and severe intrapericardial adhesions in 3 patients.

All patients were scheduled after heart team discussion,^11^ including a recommendation according to guideline indications^3^ of which coronary arteries should be grafted. Anatomic complete revascularization was defined as the successful treatment of all significant coronary lesions with a visually estimated diameter stenosis ≥50% in vessels with reference vessel diameter of ≥1.5 mm.^3^

Patients undergoing emergency procedure (ie, same-day catheterization and operation), patients with significant atheromatous disease of the ascending aorta, patients with moderate or severe aortic regurgitation, and patients undergoing reoperation were excluded.

Data are part of our internal quality assurance documentation and were retrospectively extracted from patient records and presented as mean ± SD or number (percentage). The follow-up data were collected prospectively through telephone interviews, using a structured questionnaire by a study nurse, acquisition and evaluation of medical findings, and physical examinations. Kaplan-Meier graphs were calculated with SPSS version 29.0.0.0 (IBM-SPSS Inc).

### Clinical Events

Postoperative myocardial infarction was defined as an increase in creatine kinase-MB levels within 48 hours after the procedure up to 10 times the local laboratory upper limit of normal or to five times the upper limit of normal with newly occurring Q waves in 2 contiguous leads or a new persistent left bundle branch block. This definition is in line with the Fourth Universal Definition of Myocardial Infarction of the Society for Cardiovascular Angiography and Interventions.^12^

Postoperative stroke was defined according to the updated definition of stroke for the 21st century from the American Heart Association and American Stroke Association. Stroke was characterized as a neurological deficit attributed to an acute focal injury of the central nervous system by a vascular cause, including cerebral infarction, intracerebral hemorrhage, and subarachnoid hemorrhage.^13^ A composite of all-cause-mortality, myocardial infarction, repeat revascularization, and stroke was defined as major adverse cardiac and cerebrovascular events (overall MACCE).

### Preoperative Evaluation

All patients underwent computed tomography angiography in addition to the standard institutional preoperative examinations to screen the ascending aorta, the aortic arch, and major arterial branches, especially the iliac and femoral vessels, for atherosclerotic disease and anatomical abnormalities. Quality and usability of the radial artery (RA) were evaluated with ultrasound, Doppler ultrasound, and with the Allen test.

### Anesthesia

Standard cardiac anesthesia techniques (IV sufentanil 0.5 μg/kg/hour, etomidate 0.25 mg/kg, pancuronium 0.1 mg/kg, sevoflurane, and propofol 3 mg/kg/hour) were used for induction and maintenance of anesthesia. All patients were intubated with a single-lumen endotracheal tube. Invasive monitoring was performed with standard arterial and venous lines. In all patients transoesophageal echocardiography was performed to assist positioning the guidewires for femoral arterial and venous cannulation.

### Surgical Technique

Lately, the surgical technique of total coronary revascularization via left anterior minithoracotomy was described in detail.^9^^,^^10^ The operations were performed in supine position. Endoscopic RA harvesting was performed using a reusable retractor (Bisleri Model; Karl Storz) and a bipolar radiofrequency vessel sealing system (LigaSure; Medtronic). Saphenous vein grafts (SVG) harvest was performed in an atraumatic fashion under direct surgical vision or also by using a reuseable retractor (Bisleri Model) and a bipolar radiofrequency vessel sealing system (LigaSure). Grafts were stored in a preservation solution using iron chelators (TiPROTEC; Dr Franz Köhler Chemie GmbH).

Through an anterior minithoracotomy of about 8 cm in the fourth intercostal space the chest was opened and a retractor (Small Thoracotomy Retractor; Delacroix-Chevalier) was inserted. Left internal thoracic artery (LITA) was identified and harvested under direct surgical vision as a pedicle proximally beyond the origin of left thoracic vein and distally up to the bifurcation using long conventional surgical instruments (35 cm DeBakey forceps and 15 cm electrocautery blade) and an intercostal ITA self-retraining special retractor (MIDAccess IMA Retractor, Delacroix-Chevalier) with different sizes of blades. To be able to harvest the entire length of the LITA, we first inserted the retractor distally and then proximally (Video 1).

400 U/kg heparin was administered intravenously. Peripheral arterial cannulation was performed via femoral (n = 18) artery or right axillary (n = 374) artery (16/18/20 Fr OptiSite Arterial Perfusion Cannula; Edwards Lifesciences). Percutaneous venous cannulation was achieved through the common femoral vein. A venous cannula (23 Fr Bio-Medicus; Medtronic) was placed in the right atrium guided by transoesophageal echocardiography. In case of body surface area >2.0 m^2^, an additional venous cannula was inserted in the jugular vein (15/17 Fr Bio-Medicus) to enhance the venous return. Vacuum-assisted venous return was routinely used during CPB to improve heart decompression. During CPB, patients were kept normothermic. The ascending aorta was encircled with a tape and a cannula (11 Fr DLP; Medtronic) was placed to apply cardioplegia and to vent the left ventricle (Video 1).

**Figure 1:**
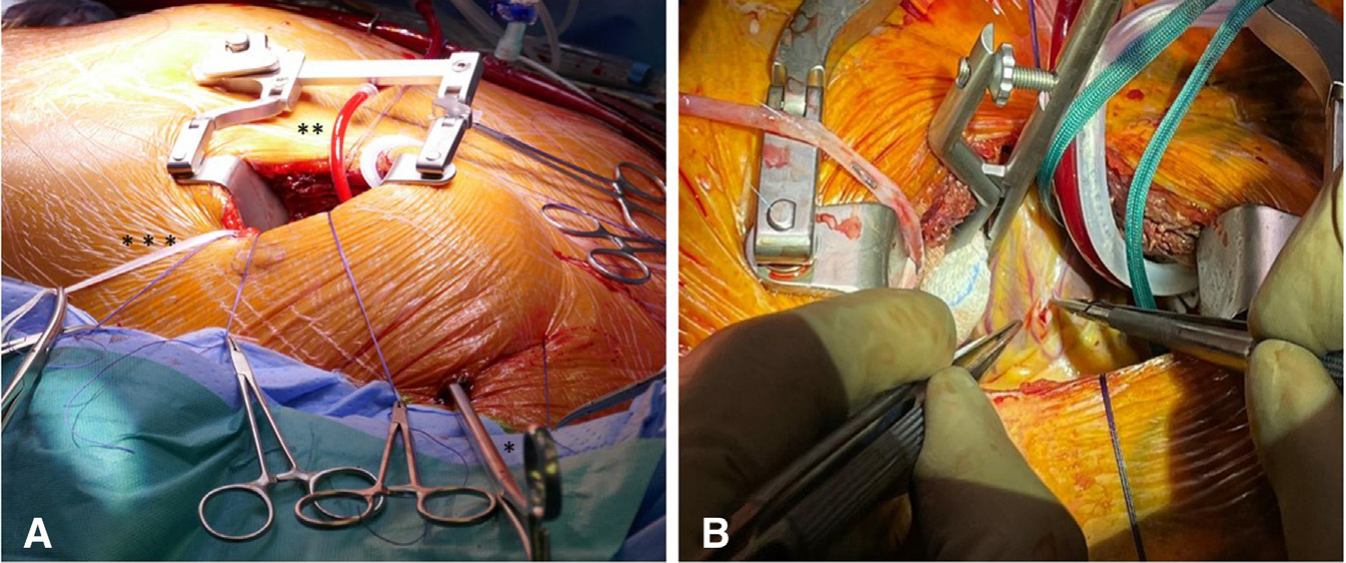
Left anterior minithoracotomy (fourth intercostal space) preparation and incision of coronary arteries. ∗Transthoracic cross-clamp, ∗∗cardioplegia line, ∗∗∗aortic sling.

Aortic crossclamp was performed using a transthoracic aortic clamp (ValveGate DeBekay; Geister), introduced through a separate small skin incision in the anterior axillary line at the level of the second intercostal space (Video 1). Diastolic cardiac arrest was induced with infusion of antegrade cold blood cardioplegia (Dr Franz Köhler Chemie GmbH) and maintained with intermittent cold reinfusion every 15 to 20 minutes (Figure 1, *A*).

After the heart was arrested and decompressed, left pulmonary veins and inferior vena cava were encircled with tapes. By pulling on these tapes in combination with rotation of the heart, the ascending aorta as well as all coronary territories could be reached by reducing the distance from skin incision of the small anterior lateral minithoracotomy to coronary arteries to <10 cm. In this way, coronary artery target sites could be exposed for manual palpation and assessment. Moreover, a stable exposition for preparation, performing of the anastomoses, and conventional manual knotting of all anastomotic sutures were possible (Video 1). Coronary anastomoses were performed with standard anastomotic technique of running 8-0 polypropylene sutures and with usual coronary surgical instruments, starting with the right coronary artery (RCA) and the circumflex artery (LCX). The LITA was anastomosed as in situ graft to the left anterior descending artery (LAD). Then the RA and/or SVG conduit was anastomosed to the LITA as composite *T*-graft or *Y*-graft or to the ascending aorta (Figure 1, *B*). All grafts were checked using transit time flow measurement (QuickFit TTFM; Medistim).

### Ethical Standards and Consent Statement

The Institutional Review Board or equivalent ethics committee of the Landesärztekammer Hessen approved the study protocol and publication of data (TEMP742237-evBO; December 28, 2021). The patients provided informed written consent for the publication of the study data.

## Results

The study group consisted of 392 consecutive, nonemergency patients (345 men; age 67.0 ± 9.9 years [range, 32-88 years]) including octogenarians (12.5%), obese patients up to 140 kg (body mass index > 35, 8.9%), patients with severe left ventricular dysfunction ejection fraction (<30%, 7.9%), patients with a recent (within the last 7 days) non-ST-elevation myocardial infarction (41.6%), and patients with an increased European System for Cardiac Operative Risk Evaluation 2 score^14^ above 4 (20.9%). Mean European System for Cardiac Operative Risk Evaluation 2 score was 2.9 ± 2.8. All patients had multivessel coronary disease, of those 32.9% having a left main stem stenosis. Baseline and clinical parameters are given in Table 1.

**Table 1:** Baseline and clinical parameters (N = 392)

Baseline and clinical parameters	Result
Age (y)	67.0 ± 9.9 (32-88)
≥80	49 (12.5)
Male	345 (88.0)
BMI	28.4 ± 4.5 (18.0-42.6)
>35	35 (8.9)
Diabetes mellitus	137 (34.9)
Chronic lung disease	67 (17.1)
Smoker	94 (24.0)
Peripheral arterial disease	137 (34.9)
Prior stroke	14 (3.6)
EuroSCORE2 (%)	2.9 ± 2.8 (0.4-29.6)
>4%	82 (20.9)
LVEF (%)	49.1 ± 9.8 (15-65)
<30%	31 (7.9)
2-vessel disease	88 (22.4)
3-vessel disease	304 (77.6)
Left main stenosis >50%	129 (32.9)
Recent NSTEMI	163 (41.6)
Prior PCI	99 (25.3)

Values are presented as mean ± SD (Minimum – Maximum) or n (%). *BMI*, Body mass index; *EuroSCORE*, European System for Cardiac Operative Risk Evaluation; *LVEF*, Left ventricular ejection fraction; *NSTEMI*, non-ST-elevation myocardial infarction; *PCI*, percutaneous coronary intervention.

Grafts used were LITA in 99.0%, RA in 70.4% and SVG in 57.4%. A total of 70.4% of all patients received at least 2 arterial grafts. Total arterial revascularization was achieved in 41.6% and multiple arterial revascularization was achieved in 28.8% of all patients. On average, 3.0 anastomoses per patient were performed, with a minimum of 2 and a maximum of 5 anastomoses. The LAD territory was grafted in 98.7% (in 99.5% with arterial grafts), the LCX territory in 91.6% (in 72.4% with arterial grafts), and the RCA territory in 70.9% (in 34.1% with arterial grafts) of patients. Complete anatomical revascularization was achieved in 95.1%. Operative data are given in Table 2.

**Table 2:** Operative data (N = 392)

Operative data	Result
Conduits	
LITA	388 (99.0)
Radial artery	276 (70.4)
Saphenous vein	225 (57.4)
Revascularized territory (proportion arterial grafts)	
LAD	387 (98.7)
Of these arterial	385 (99.5)
LCX	359 (91.6)
Of these arterial	260 (72.4)
RCA	278 (70.9)
Of these arterial	98 (34.1)
No. of distal anastomoses	3.0 ± 0.8 (2-5)
Complete anatomical revascularization	373 (95.1)
Duration (min)	
CPB	161 ± 41 (52–313)
Aortic crossclamping	100 ± 32 (22-255)
Operation	334 ± 73 (145-705)

Values are presented as mean ± SD (Minimum – Maximum) or n (%). *LITA*, Left internal thoracic artery; *LAD*, left anterior descending; *LCX*, left circumflex artery; *RCA*, right coronary artery; *CPB*, cardiopulmonary bypass.

**Figure 2:**
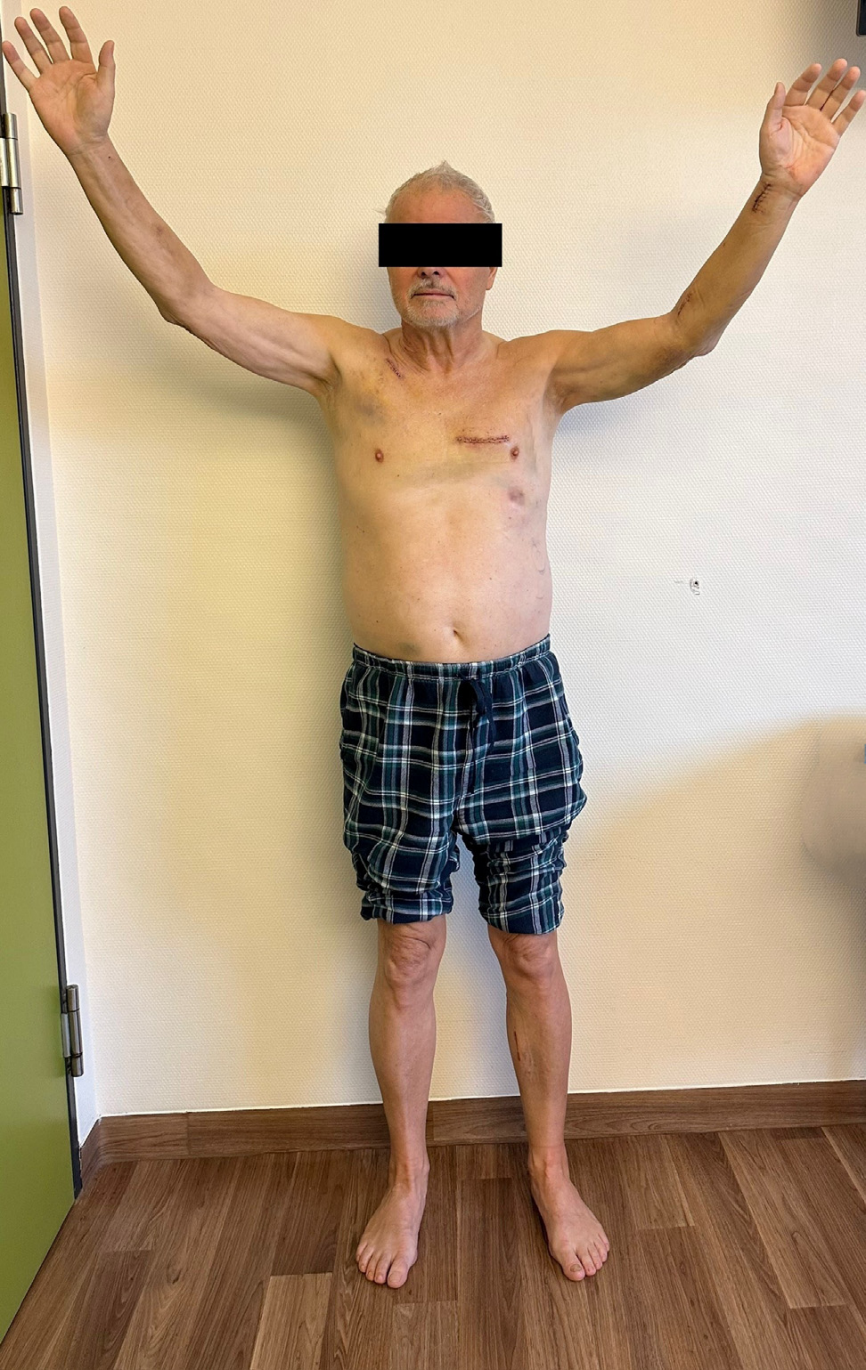
Patient aged 67 years on third postoperative day after quadruple bypass using Total coronary revascularization via anterior thoracotomy technique with endoscopic radial and saphenous vein harvesting.

Avoiding sternotomy allowed immediate mobilization (Figure 2). 67% of patients left the intensive care unit within the first postoperative day. In-hospital mortality was 1.3%, with 2 patients dying from noncardiac complications (pneumonia/respiratory failure and bowel obstruction). In-hospital myocardial infarction was 0.5%, repeat revascularization was 1.0%. One patient experienced a perioperative stroke (0.3%) with minor clinical impairment. Postoperative adverse events and outcome are given in Table 3.

**Table 3:** In-hospital outcome (N = 392)

In-hospital outcome	Result
In-hospital mortality	5 (1.3)
Myocardial infarction	2 (0.5)
Repeat revascularization (PCI)	4 (1.0)
Stroke	1 (0.3)
In-hospital MACCE	9 (2.3)
Length of ICU stay (d)	10.8 ± 8.2 (2-71)
1	263 (67)
2-4	102 (26)
Other complications	
Revision due to bleeding	28 (7.1)
Delirium	39 (10.0)
Pneumonia	6 (1.5)
New onset of atrial fibrillation	44 (11.2)
Total wound healing complications	8 (2.0)
Superficial	6 (1.5)
Deep	2 (0.5)
Empyema	1 (0.3)
Chest wall hernia	1 (0.3)
Acute renal failure requiring dialysis	7 (1.8)

Values are presented as mean ± SD (Minimum – Maximum) or n (%). *PCI*, Percutaneous coronary intervention; *MACCE*, major adverse cardiac and cerebrovascular events; *ICU*, intensive care unit.

**Figure 3:**
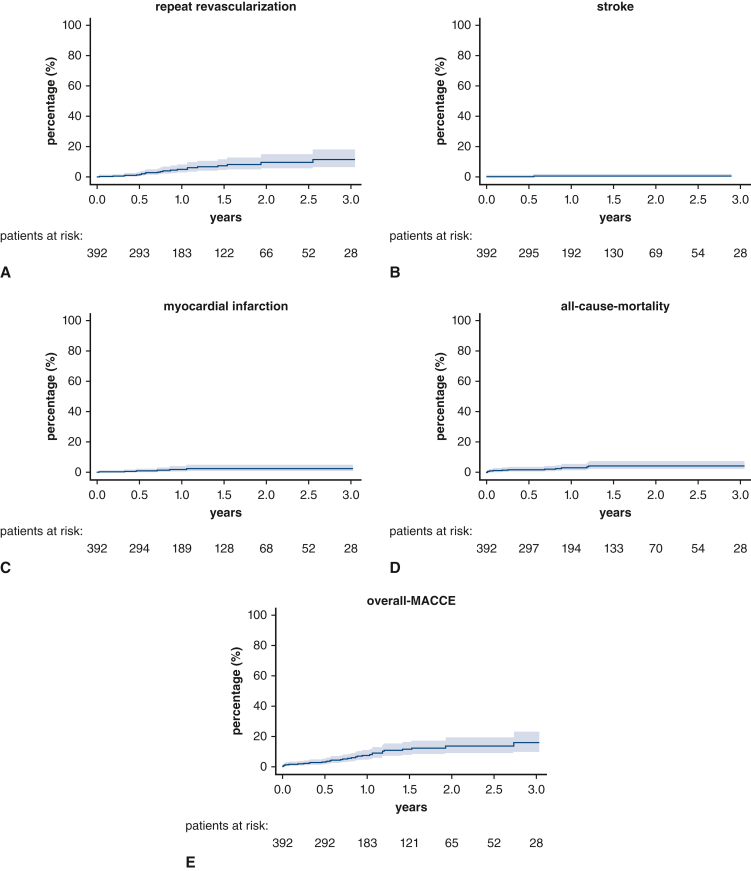
Kaplan-Meier graphs of major adverse cardiac and cerebrovascular events (MACCE) (confidence limits 95%). A, Repeat revascularization. B, Stroke. C, Myocardial infraction. D, All-cause-mortality. E, Overall MACCE.

**Figure 4:**
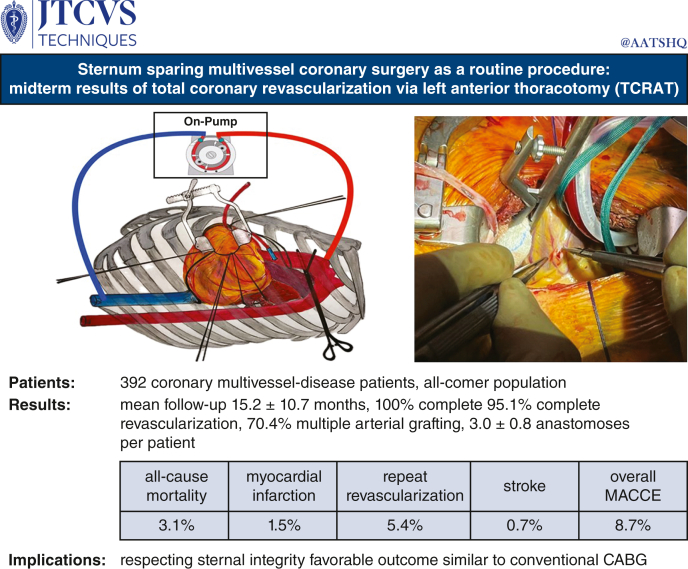
Graphical abstract.

Mean follow-up was 15.2 ± 10.7 months (range, 0.06-39.5 months) and was completed to 100%. Median of our follow-up was 11.8 months (interquartile range, 13.9 months [6.1-20.2 months]). During follow-up, all-cause-mortality was 3.1%, postoperative myocardial infarction was 1.5%, and postoperative stroke was found in 0.7% of patients. Repeat revascularization was 5.4%. Of these, 4 patients (1%) were planned for hybrid procedures and underwent postoperative percutaneous coronary intervention. Overall MACCE during the observed follow-up was 8.7%. Follow-up data are provided in Table 4, in Figure 3 and in Figure 4.

**Table 4:** Midterm follow-up data (N = 392)

Midterm outcome	Result
All-cause-mortality	12 (3.1)
Myocardial infarction	6 (1.5)
Repeat revascularization (PCI)	21 (5.4)
Of these planned procedures	4 (1.0)
Stroke	3 (0.7)
Overall MACCE during follow-up	34 (8.7)

Values are presented as n (%). *PCI*, Percutaneous coronary intervention; *MACCE*, major adverse cardiac and cerebrovascular events.

## Discussion

The present study demonstrated that sternum sparing CABG using the TCRAT technique enables complete, predominantly arterial revascularization and was safe as a routine procedure in a nonselected patient population. In-hospital and midterm results were similar to contemporary conventional total sternotomy CABG.

Long-term benefit from surgical compared with interventional myocardial revascularization is most pronounced in patients with multivessel disease with diffuse coronary disease (high Synergy Between Percutaneous Coronary Intervention With Taxus and Cardiac Surgery [SYNTAX] score), diabetes mellitus, and with reduced left ventricular function.^3^ In addition, nowadays CABG patients often experience high comorbidity and surgical risk. Although these patients often were regarded as not well suited for minimally invasive techniques,^7^^,^^15^^,^^16^ we included patients with diffuse coronary disease requiring complex coronary surgery, with very low left ventricular ejection fraction, and with high surgical risk. Also, we did not regard massive obesity or compromised lung function as contraindications for the presented TCRAT technique. Thus, we present a representative, contemporary all-comer CABG population.

The results of our study are in the range with 1-year-follow-up outcomes reported in the important myocardial revascularization trials of the recent decades^17^; for example, SYNTAX^18^ and the Fractional Flow Reserve versus Angiography for Multivessel Evaluation (FAME 3)^19^ trial. However, more patients with a higher surgical risk profile were included in our study. After 12 months, the SYNTAX trial reported a MACCE rate between 12.9% and 15.5%, depending on the SYNTAX score. In the FAME 3 trial, 1-year outcomes showed a MACCE rate of 6.9%. Thus, the 15 months rate of 8.7% MACCE reported in our study favorably compares to these trials of conventional bypass surgery with sternotomy. Likewise, the rate of repeat revascularization in our study (15 months, 5.4%) is in the range of what was reported in the most recent FAME 3 trial (12 months, 3.9%). In this context, it is worth mentioning that in our study the reported rate of repeat revascularization also included planned percutaneous coronary intervention as part of hybrid procedures.

Another recently published multicenter registry study (DuraGraft Registry) prospectively examined the clinical outcome after contemporary conventional CABG.^20^ In patients undergoing isolated CABG, the rate of MACCE after 1 year was 7.8%. Reported rates for all-cause-mortality, myocardial infarction, and stroke were 4.4%, 2.0%, and 1.9%, respectively. These results are consistent with our findings, although the surgical risk profile in our patient population and the percentage of multiple arterial grafting were considerably higher.

Due to the pathophysiological concept of surgical collateralization, the effect of CABG in stable coronary multivessel disease is based on bypassing diseased proximal coronary artery segments with a vascular conduit to prevent future myocardial infarction, and thus prolonging life.^21^ Therefore, key issues for a long-term success of CABG are a complete anatomical revascularization, optimal performance of anastomoses, and durability of used conduits.^3^

To achieve a complete anatomical revascularization in patients with multivessel disease, all coronary arteries with a diameter exceeding 1.5 mm and a luminal reduction of 50% or more should be grafted.^3^ According to this definition, a complete anatomical revascularization was accomplished in 95.1% of patients in our study. The number of grafts performed in patients with multivessel disease often is considered as an indirect parameter for the completeness of revascularization.^22^ The average number of grafts performed in the present study was 3.0, which compares well to the average number of 3.0 grafts reported for conventional CABG in the German Heart Surgery Report 2020.^23^ However, it clearly exceeds the average number of 2.4 grafts in patients operated off-pump reported in the same registry^23^ as well as the average number of grafts reported in MICS CABG.^6^^,^^15^^,^^16^

Stable exposition of a silent operative situs enables optimal creation of the coronary anastomoses, which were performed with standard anastomotic technique of running 8-0 polypropylene sutures and with usual coronary surgical instruments. With the heart arrested and emptied, the working space inside the pericardium becomes much larger, whereas hemodynamics remain stable, thus allowing the surgeon to encircle the left pulmonary veins and the inferior vena cava with tapes. Traction on these tapes and rotating the heart enabled to reach all left ventricular territories by reducing the distance from skin incision to coronary arteries significantly to <10 cm even in patients with obesity. Coronary artery target sites, especially the RCA and LCX territories at the lateral and inferior left ventricular wall could be exposed.^8^ This is evident in our study, with a high percentage of patients undergoing revascularization of the RCA territory in 70.9% and of the LCX territory in 91.6%. Again, this was clearly more often compared to off pump MICS CABG,^6^^,^^15^^,^^16^ ranging from 10% to 24% for the RCA and from 33% to 84% for the LCX.

It has been consistently shown that arterial grafts exhibit lower long-term occlusion rates compared with venous grafts.^24^^,^^25^ Therefore, beside the use of the LITA graft to the LAD, the additional use of a second arterial graft is recommended in recent revascularization guidelines.^3^ Such a bypass material strategy has been applied in a majority of patients in the present study with 70% multiple arterial grafting.

Attributed to a smaller surgical incision and an increased technical complexity, it may be of concern that duration of the operation, CPB and aortic crossclamping was clearly longer than that known from routine CABG. Moreover, the need to carry out some surgical steps in sequence and not simultaneously; for example, harvesting of RA and ITA, resulted in a prolonged operation time. However, this was even rewarded by a shorter intensive care unit stay and faster discharge in comparison to that of our patients undergoing routine CABG. Additionally, we believe more experience will allow us to shorten the procedure distinctly, as has been shown previously for newly introduced surgical techniques.^15^

Aortic manipulation and CPB, especially when performed with peripheral arterial cannulation and retrograde perfusion, may be associated with an increased stroke rate. To minimize this risk, we routinely performed a preoperative computed tomography scan to identify patients with atherosclerotic disease.^26^ In case of atherosclerosis distal to the ascending aorta, a more central cannulation via the right subclavian artery was preferred. As consequence of a stroke in a patient perfused from femoral arterial cannulation, we changed to a strictly central cannulation via the right subclavian artery and did not observe any stroke in the consecutive series of patients. In case of evidence of atherosclerotic disease of the ascending aorta, alternative no-touch aortic CABG techniques should be preferred. Whether such algorithm is effective in minimizing stroke risk remains to be investigated in further studies. However, the 0.3% stroke rate observed in our series is clearly below the 30-day stroke rate reported for contemporary standard CABG of about 1.5%^1^^,^^19^ and comparable to 30-day stroke rate of anaortic off-pump CABG of 0.4%.^27^

The main advantage of the TCRAT technique, when compared with standard CABG, obviously results from the avoidance of sternotomy.^28^ The risks of superficial or deep sternal wound infections, as well as sternal instability, are completely eliminated. Enhanced thoracic stability, reduced wound size, and reduced wound infection risk associated with a minimally invasive thoracotomy approach have already been shown to result in accelerated early recovery and return to normal physical activity in different cardiac surgery procedures.^29^^,^^30^ Currently, an ongoing multicenter randomized controlled trial compares the quality of life and recovery between nonsternotomy MICS CABG and sternotomy CABG.^31^ Nevertheless, respecting sternal integrity might considerably improve both patients' and physicians’ acceptance of surgical myocardial revascularization.

### Study Limitations

There are several limitations of our study. The present study is a single-center study to investigate a new surgical technique and its results. It should be noted that it includes the learning curves of 2 surgeons (H.D. and C.S.). Although only conventional surgical techniques are used, surgeons have to adapt to some new aspects operating through a smaller incision with a different view and perspective of the heart pulled by the tapes; for example, we have learned that color marking of the grafts is helpful to avoid twisting them. Of note, duration of the TCRAT procedure is longer than conventional CABG.^9^^,^^10^ However, the price of longer surgical time pays off into a short length of intensive care unit stay, elimination of sternal wound infections, and superior patient comfort with rapid mobilization due to better maintenance of mechanical resilience of the thorax.

Despite reflecting a broad range of all-comer patients, there is a certain patient selection because patients who showed severe calcifications of the ascending aorta or need for emergency surgery were not treated with TCRAT. Although we included urgent patients with acute coronary syndromes, patients with same-day emergency surgery were excluded to prepare operating room resources.

Furthermore, mean follow-up of 15 months was only midterm. Longer follow-up periods are necessary to further assess the role of this new surgical approach in coronary surgery. However, the midterm outcome of the present study is favorable and similar to contemporary CABG; therefore, it is likely that long-term results of TCRAT remain propitiously, especially with multiple arterial grafting because it has been proven in many studies with conventional CABG.^17^

## Conclusions

This is the first report of midterm follow-up in unselected patients undergoing TCRAT. This sternum-sparing approach allows complete and predominant arterial coronary revascularization in a broad majority of multivessel disease with favorable in-hospital and midterm outcomes. Long-term results remain to be investigated.

### Webcast

You can watch a Webcast of this AATS meeting presentation by going to: https://www.aats.org/resources/sternum-sparing-multi-vessel-c-7202.

**Figure:**
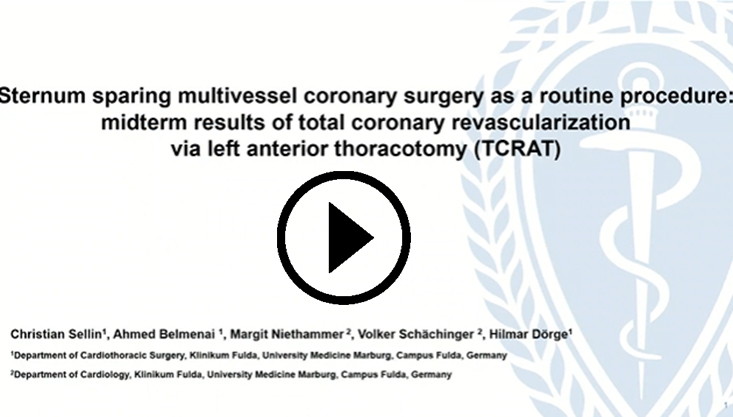

## Conflict of Interest Statement

The authors reported no conflicts of interest.

The *Journal* policy requires editors and reviewers to disclose conflicts of interest and to decline handling or reviewing manuscripts for which they may have a conflict of interest. The editors and reviewers of this article have no conflicts of interest.

## Supplementary Data

Video 1 Technical aspects of minimally invasive coronary artery bypass grafting: Total coronary revascularization via anterior thoracotomy technique. Video available at: https://www.jtcvs.org/article/S2666-2507(24)00239-6/fulltext.
