# Supplementary figures and images for: Sternum-sparing multivessel coronary surgery as a routine procedure: Midterm results of total coronary revascularization via left anterior thoracotomy
Source: JTCVS Tech. 2024 Jun 3;26:52–60. doi: 10.1016/j.xjtc.2024.05.018 (PMC11329208; doi:10.1016/j.xjtc.2024.05.018)

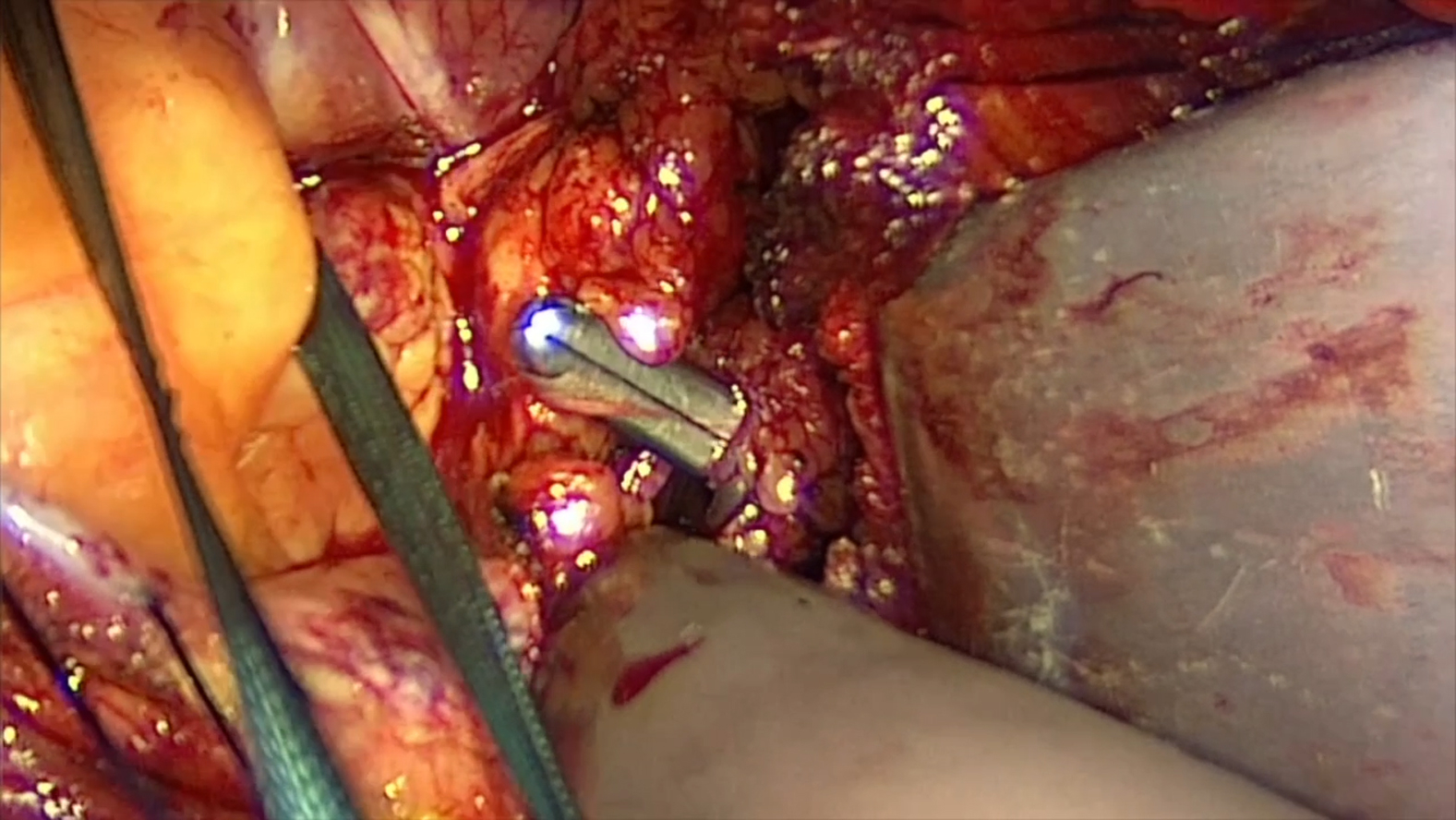

Supplement: Video 1 — Technical aspects of minimally invasive coronary artery bypass grafting: Total coronary revascularization via anterior thoracotomy technique. Video available at: https://www.jtcvs.org/article/S2666-2507(24)00239-6/fulltext. [file fx2.jpg]
